# JSCC-Cast: A Joint Source Channel Coding Video Encoding and Transmission System with Limited Digital Metadata

**DOI:** 10.3390/s21186208

**Published:** 2021-09-16

**Authors:** Jose Balsa, Óscar Fresnedo, José A. García-Naya, Tomás Domínguez-Bolaño, Luis Castedo

**Affiliations:** CITIC Research Center, Department of Computer Engineering, University of A Coruña, 15008 A Coruña, Spain; oscar.fresnedo@udc.es (Ó.F.); jagarcia@udc.es (J.A.G.-N.); tomas.bolano@udc.es (T.D.-B.); luis.castedo@udc.es (L.C.)

**Keywords:** analog processing, analog video encoding, analog video transmission, joint source-channel encoding

## Abstract

This work considers the design and practical implementation of JSCC-Cast, a comprehensive analog video encoding and transmission system requiring a reduced amount of digital metadata. Suitable applications for JSCC-Cast are multicast transmissions over time-varying channels and Internet of Things wireless connectivity of end devices having severe constraints on their computational capabilities. The proposed system exhibits a similar image quality compared to existing analog and hybrid encoding alternatives such as Softcast. Its design is based on the use of linear transforms that exploit the spatial and temporal redundancy and the analog encoding of the transformed coefficients with different protection levels depending on their relevance. JSCC-Cast is compared to Softcast, which is considered the benchmark for analog and hybrid video coding, and with an all-digital H.265-based encoder. The results show that, depending on the scenario and considering image quality metrics such as the structural similarity index measure, the peak signal-to-noise ratio, and the perceived quality of the video, JSCC-Cast exhibits a performance close to that of Softcast but with less metadata and not requiring a feedback channel in order to track channel variations. Moreover, in some circumstances, the JSCC-Cast obtains a perceived quality for the frames comparable to those displayed by the digital one.

## 1. Introduction

Video system research and developmental studies are currently focused on digital systems due to their great adaptability, although there are a number of situations in which analog encoding and transmission video systems can outperform their all-digital equivalents:

- Analog encoding and transmission systems are capable of adapting to the channel conditions without any prior knowledge. By contrast, all-digital video systems require either certain channel information provided by a feedback channel or the transmission of a large amount of redundancy in order to ensure successful reception regardless of the channel conditions.
- All-digital video systems guarantee error-free transmissions above a certain channel quality, keeping fixed the received video quality, hence, wasting bandwidth due to redundancy. In case the channel quality is not sufficient to guarantee an error-free transmission, the video quality will be significantly degraded, and even temporal drops in the video sequence will occur due to the loss of complete video frames.
- In a wireless-multicast scenario, an all-digital system has to target the worst of the receivers or broadcast several video layers simultaneously. The required redundancy to combat low-quality channels will penalize those receivers with high-quality channels. Notice that in the case of multiple video layers, the bandwidth will also be reduced. By contrast, analog encoding and transmission video systems send exactly the same information to all the receivers regardless of their channel status. The quality of the image received will depend on the channel quality experienced by each receiver.
- Video visualization on different types of fixed and mobile devices is growing every year [1]. This makes it necessary to develop advanced video coding and transmission systems. In particular, with the growth of the Internet of Things, the use of simple microprocessors with very low power consumption is expanding, thus requiring systems with ultra-low complexity and computational load [2,3].

In summary, analog encoding and transmission video systems are simpler than their all-digital equivalents since the transmission is the same for all receivers, and no feedback channel is required.

This paper investigates a functional scheme in order to reduce the bandwidth required by raw video transmission over channels with limited capacity while keeping an acceptable video quality level. In order to address this objective, JSCC-Cast is proposed, which is a low-complexity scheme for encoding analog video with negligible digital metadata and with special emphasis on its suitability for Internet of things (IoT) systems.

JSCC-Cast extends our previous work on coding and transmission of still images based on analog joint source channel coding (AJSCC) techniques [4]. In order to take advantage of video spatial and temporal redundancy, first, the video sequence is split into blocks, applying to them a discrete cosine transform (DCT). Next, the transmitted coefficients are protected against noise with an analog mapping. Finally, prior to its transmission, the symbols are shuffled with a Hadamard matrix in order to equalize the frame quality. At reception, the inverse operations are carried out to recover the blocks of pixels and to reconstruct the original video sequence.

### Contributions of the Paper

The contributions of this work can be summarized as follows:

1. JSCC-Cast, a comprehensive analog video encoding and transmission system, is proposed which requires a minimum amount of metadata to reconstruct the video. Since the corruption of metadata during the transmission makes video decoding impossible, it is crucial to properly protect such metadata, resulting in a reduction in the available bandwidth for video data. JSCC-Cast is also designed to provide a video quality comparable to other similar alternatives with high compression levels and minimum computational cost and delay.
2. A detailed analysis and design of the different components of JSCC-Cast is performed. This analysis allows us to evaluate the impact of several design parameters on the system performance and the quality of the resulting decoded video.

## 2. State of the Art

The following is a review of the main existing approaches relative to video encoding and transmission.

### 2.1. All-Digital Video Systems

Although all-digital video systems usually offer the best transmission characteristics for any media due to the great research progress over the last decades, the analog encoding and transmission of video in certain scenarios offers important advantages.

One such scenario is wireless multicast transmission in which the receivers have different channel quality levels. In this case, digital systems adapt their transmission rate to the worst receiver, thus penalizing the receivers that could obtain higher quality. Video transmission over time-variant channels is also problematic for all-digital systems since they have to constantly adapt their transmission rates. This imposes severe requirements on the video encoder, especially if it has to operate in real time. Notice that wireless digital transmission systems rely on strategies such as adaptive modulation and coding to match the transmission rate relative to the channel capacity. This requires a feedback link to inform the transmitter about the channel quality observed at the receiver. Although this approach performs well in quasi-static scenarios, video transmission over highly time-variant channels still remains a challenge since the throughput noticeably deviates from the channel capacity [5].

There are proposals in the literature that seek to mitigate this problem by transmitting digital video sequences with different bandwidth ratios and encoding them in several video layers [6,7]: A base layer with highest protection against channel distortions and meeting the lowest quality requirements is followed by layers encoded on top with a lower level of protection and provide increasing quality. Typically, these systems are complex in terms of implementation and do not take advantage of all the available bandwidth. Thus, they deliver video sequences with quality levels lower than the possibilities of the wireless channel.

### 2.2. Hybrid Video Systems

Hybrid video systems combine both analog and digital strategies in order to obtain simpler schemes. Hybrid approaches benefit from the advantages of the analog systems in which the transmitted symbols are always received, although they are distorted with noise. Therefore, in multi-cast transmission, the receivers decode the video with a quality inversely proportional to the channel noise level.

The hybrid video system of reference in the literature is the so-called Softcast [8], which makes use of a three-dimensional (3D) DCT followed by a decimation according to the target transmission rate. The resulting analog data symbols are sent following an analog joint source channel coding (JSCC) scheme, and the corresponding metadata is digitally encoded prior to its transmission. The amount of metadata, which in the case of Softcast depends on the content of the video sequence, is precisely its main disadvantage because the video decoding procedure fully depends on the perfect reception of the metadata. In order to avoid this situation, metadata is transmitted with high redundancy levels at speeds much lower than the channel capacity, hence wasting available bandwidth. The main advantages of Softcast with respect to all-digital schemes include its higher simplicity, and its ability to exhibit image quality degradation proportional to the channel noise level.

Apart from Softcast, many other hybrid video systems have been also proposed. Most of them make use of the DCT or the wavelet transform in some of the video processing steps. They all have in common the need for a large amount of metadata, which is critical for video decoding. The following is a review of the hybrid systems that exist in the literature at present, classified according to the used transform and sorted by chronological order.

#### 2.2.1. 2D-DCT Hybrid Systems

- DCAST [9] exploits temporal redundancy through the technique of distributed source coding, making use of coset and syndrome coding. DCAST transmits an amount of metadata larger than Softcast because it has to send temporal redundancy information without errors in order to produce correct video decoding.
- The system proposed in [10] uses high-efficiency video coding (HEVC) as a base layer, and the residual is transmitted as a two-dimensional (2D)-DCT version of Softcast.
- SparseCast [11] uses compressive sensing instead of decimation of the DCT output; it does not consider the time dimension and, hence, is limited to the 2D-DCT.
- CG-Cast [12] is a Softcast-based system that employs a compressive-gradient-based image representation to describe perceptually sensitive image details. It also uses the fast Fourier transform (FFT) to determine the low-frequency data corresponding to the global and local luminance of the image.

#### 2.2.2. 3D-DCT Hybrid Systems

- ParCast [13] is a video system similar to Softcast in which the encoded symbols are simultaneously transmitted over orthogonal subchannels. ParCast is more sophisticated than Softcast because the subchannels are optimized individually and requires a feedback channel. A later version, called ParCast+ [14], utilizes a motion compensated temporal filtering (MCTF) in order to integrate temporal redundancy within a 2D-DCT and, therefore, improves performance.
- The work in [15] is a Softcast-based system optimised for the transmission over fast fading channels. This is achieved by prioritizing and altering the order of the transmitted symbols according to the power of the DCT coefficients.
- SharpCast [16] is a hybrid system that uses HEVC to send the structure of the image and a 3D-DCT to transmit the residual. A similar system is proposed in [17] using Shannon–Kotel’nikov mapping to protect the analog residue. The system proposed in [18] is also similar to SharpCast, with the advantage of not requiring perfect decoding of the digital data in order to recover the video.
- Wireless Cooperative Video Coding (WCVC) [19] is based on the idea that certain nodes repeat the source signal over wireless channels using hybrid technologies. This system is based on the H.264/AVC standard to encode the base layer and an analog system similar to Softcast for the residues of the digital system. Hence, the modulation scheme superimposes the digital and analog information so that the digital part is the basic quality layer and the analog part adds quality to the video. This system is highly complex and computationally expensive since it requires two complete analog and digital schemes.
- The system described in [20] improves the Softcast scheme by protecting the top-left region at the 3D-DCT output matrix with a digital transmission. This system exhibits a better performance at the expense of increasing the amount of digital data.
- The system described in [21] is another version of Softcast that uses Gaussian Markov random field (GMRF) to significantly reduce the amount of metadata, while maintaining high video quality.
- MCast [22] is a version of Softcast optimised to achieve high-quality video transmissions over time-frequency varying channels.

#### 2.2.3. Wavelet-Based Hybrid Systems

- WaveCast [23] employs 3D Wavelets together with a motion time filter in order to efficiently exploit the temporal redundancy. However, this information needs to be transmitted with no errors for the decoder to work properly. Thus, it requires an even larger amount of metadata than previous systems.
- The work in [24] describes a system which encodes the 2D-discrete wavelet transform (DWT) output low-frequency subband using H.264/AVC, while LH, HL, and HH are encoded with a DCT-based approach.
- Adaptive hybrid digital–analog video transmission (A-HDAVT) [25] is a fading channel oriented video transmission scheme. Some frames are selected to be transmitted digitally using H.264/AVC, while others are transmitted using an analog Haar DWT.

## 3. JSCC-Cast: Encoding and Transmission System

JSCC-Cast aims at encoding and transmitting video with low complexity, low delay, and minimum metadata information. The system is also suitable for the transmission over time-varying channels thanks to the intrinsic characteristics of the analog encoding of the video information. In addition, the objective is to obtain a comprehensive system that is competitive with hybrid alternatives and even with all-digital schemes traditionally employed in video compression. The components of the analog transmitter and receiver are described below.

### 3.1. JSCC-Cast: Analog Encoder and Transmitter

The sequence of frames corresponding to the video to be transmitted is the input to our system. We focus on grayscale frames for simplicity. The information processing can be generalized in order to color videos since similar operations are applied individually to each color component.

**Figure 1:**
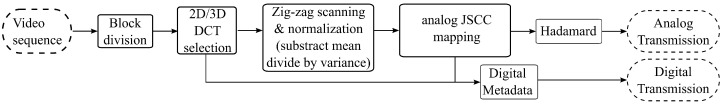
Block diagram of the JSCC-Cast encoder and transmitter.

Figure 1 plots the block diagram of the analog video encoder and transmitter of JSCC-Cast. The input video sequence is first divided into 3D arrays or blocks of pixels having the dimensions B×B×F, where *B* is the number of pixels per spatial dimension and *F* is the number of frames in the temporal dimension. Therefore, the total number of pixels per 3D array is $B^{2}F$.

In the next step, the system applies a domain transform to the 3D arrays in order to compact the signal energy into a smaller number of relevant coefficients. In the transformed domain, non-relevant information can be easily removed with a negligible impact on the video representation. Indeed, the coefficients after the transform are ordered according to their importance and then discarded or decimated from less to most important until selecting the number of coefficients required to achieve the target compression level.

The use of domain transforms and the removal of dispensable coefficients are essential for the exploitation of the spatial and temporal video redundancy for compressing video representation. As shown in [4,8], the direct analog compression of visual information in the spatial-temporal domain does not work properly due to the high amount of redundancy of this type of data.

The coefficients at the output of the transform operation are organized next into two different data streams due to its different impact on the visual quality, namely direct current (DC) and alternate current (AC) coefficients. Let $s_{j}=\left[s_{1,j},\ldots ,s_{N_{j},j}\right]$ be the *j*-th vector of transform coefficients after this reorganization. The subindex j=1 corresponds to the DC coefficients data stream, whereas j=2 identifies the AC data stream. Note that $N_{j}$ is the size of the vector $s_{j}$. Therefore, $N_{1}+N_{2}$ will be the total number of compressed symbols and the compression ratio (CR) is given by the following.

(1) $$\mathrm{CR}=\frac{B^{2}F}{N_{1}+N_{2}}.$$

Since the analog JSCC mappings employed for the encoding operation perform better with normalized inputs, the vectors $s_{j}$ are individually normalized by subtracting their mean and normalizing their variance, i.e, we have the following:

(2) $$\begin{array}{c} {\bar{s}}_{j}=\frac{s_{j}-\mu_{j}}{\sigma_{j}}, \end{array}$$

where $\mu_{j}$ and $\sigma_{j}^{2}$ are the mean and the variance of the *j*-th data stream, respectively. These parameters must be included as digital metadata because they are needed at the receiver to undo normalization.

The next step is the encoding of the transformed coefficients. This operation is carried out in the analog domain. The real-valued coefficients ${\bar{s}}_{j}\in R^{N_{j}}$ are directly transformed into the real-valued encoded symbols $x_{j}\in R^{M_{j}}$, with $M_{j}\geq N_{j}$, as follows:

(3) $$x_{j}=f_{j}\left({\bar{s}}_{j}\right),\;\;j=1,2$$

where $f_{j}\left(\cdot \right):R^{N_{j}}\to R^{M_{j}}$ is a continuous mapping function appropriate for the encoding of the symbols in the *j*-th data stream. Note that, in general, the continuous mappings can expand the dimension of the symbols to be transmitted; hence, the expansion factor is defined as follows.

(4) $$L_{j}=\frac{M_{j}}{N_{j}}\geq 1.$$

In the limiting case $L_{j}=1$, no expansion occurs since $M_{j}=N_{j}$. The total number of analog encoded symbols is, hence, $L_{1}N_{1}+L_{2}N_{2}$, and this determines the following bandwidth ratio:

(5) $$\begin{array}{c} BW_{r}=\frac{L_{1}N_{1}+L_{2}N_{2}}{B^{2}F}, \end{array}$$

which is the quotient between the number of transmitted symbols and the number of input pixels.

Following our previous work in [4], we consider parametric mappings, which are able to adjust the amount of channel redundancy depending on the type of coefficients to be encoded, and hence providing different levels of symbol protection against channel distortions. In this sense, the analog encoding aims at improving the quality of the received information while avoiding an excessive amount of redundancy. Towards this aim, two of the analog JSCC mappings already evaluated in [4] for still images are also considered for the analog encoding of video sequences in JSCC-Cast due to their flexibility, low complexity, and negligible delay.

In the first case, a linear mapping is considered where the input symbols are simply scaled before transmission by a linear factor, i.e., we have the following:

(6) $$x_{j}=f_{j}\left({\bar{s}}_{j}\right)=\sqrt{\lambda }\;{\bar{s}}_{j},$$

where λ is the scaling parameter. Note that when using a linear mapping, the expansion factor is $L_{j}=1$, i.e., analog encoding does not introduce redundancy.

In the second case, we considered orthogonal spherical codes based on the exponential chirp modulation [26], which is a type of nonlinear mapping with an integer expansion factor $L_{j}\geq 2$. According to such codes, the scalar symbol ${\bar{s}}_{i,j}$, i.e., the *i*-th entry of the the normalized input vector ${\bar{s}}_{j}$ given by (2), is mapped to the encoded symbol vector $x_{i,j}\in R^{L_{j}}$ as follows:

(7) $$\begin{array}{cc} x_{i,j}= & f_{j}\left({\bar{s}}_{i,j}\right)=\Delta [\cos\left(2\pi {\bar{s}}_{i,j}\right),\sin\left(2\pi {\bar{s}}_{i,j}\right),\cos\left(2\pi \alpha {\bar{s}}_{i,j}\right),\sin\left(2\pi \alpha {\bar{s}}_{i,j}\right),\ldots ,\\ \; & \cos\left(2\pi \alpha^{(L_{j}/2)-1}{\bar{s}}_{i,j}\right),\sin\left(2\pi \alpha^{(L_{j}/2)-1}{\bar{s}}_{i,j}\right)], \end{array}$$

where Δ and α are parameters of the mapping. The resulting encoded symbols $x_{i,j}$ are next stacked into the following vector.

(8) $$x_{j}=\left[x_{1,j},\ldots ,x_{N_{j},j}\right].$$

As observed, the nonlinear mapping given by (7) consists of sinusoidal functions with different frequencies. The parameter α determines the frequencies of these sinusoidal functions, while Δ is set to $\sqrt{2}$ to ensure that the average power of the encoded symbols is normalized. The parameter α should be adjusted depending on the channel conditions, but this would result in an increase in the amount of metadata to be sent to the receiver. However, as observed in [4], performance degrades gracefully when using non-optimal parameters; thus, using fixed values for α and Δ will have a small impact on the system performance.

Note that a different analog mapping could be applied to each coefficient stream ${\bar{s}}_{j}$, with a different level of protection (and a different expansion factor) depending on the type of coefficients or even on the channel conditions. This feature of JSCC-Cast represents an interesting advantage for properly balancing the bandwidth rate and the video quality.

This same procedure is repeated for all the B×B×F 3D arrays corresponding to the selected *F* frames of the input video sequence, stacking all the resulting encoded symbols into a single vector x.

An important issue is that the transmit power is not uniformly distributed among the encoded symbols; therefore, the channel noise can distort differently the distinct parts of the video frames during the reconstruction procedure. This same problem was identified when designing the Softcast system [8] and solved by applying an fast Hadamard transform (FHT) to the encoded symbols [27,28,29]. This redistributes the instantaneous power in a more homogeneous manner over the transmitted symbols, and it ensures that the quality of the decoding procedure gracefully degrades as information losses increase.

The FHT is then applied to the encoded symbols as follows:

(9) $$Y=\sqrt{P}\cdot H_{n}\left(X\right),$$

where *P* represents the available transmit power and $H_{n}\left(\cdot \right)$ is the operator of the FHT of order $n=2^{k}$, which is applied column-wise to the $X\in R^{n\times m}$ matrix containing all the encoded symbols in x and with a zero padding in order to adjust it relative to the Hadamard order. The result of this operation is the matrix of encoded symbols Y, which is also of dimensions n×m.

Note that, for each 3D array, the system requires the knowledge of the two pairs of mean and variance values for the AC and DC symbols, respectively. This metadata is assumed to be transmitted digitally. It is first compressed by means of Huffman coding and next encoded with a convolutional code of rate 1/2 for error protection. This guarantees an error-free reception of the metadata in the considered channel scenarios.

### 3.2. AWGN Channel

The real-valued analog-encoded symbols generated at the transmitter are assumed to be sent over an additive white Gaussian noise (AWGN) channel. Hence, the received symbols can be expressed as follows:

(10) $$\hat{Y}=Y+N,$$

where $N\in R^{n\times m}$ represents the AWGN matrix for which its entries are i.i.d. and follow a Gaussian distribution such that $n_{i,j}∼N\left(0,n_{0}\right)$, where $n_{0}$ is the channel noise variance. Without the loss of generality, we will assume that $n_{0}=1$. Hence, the channel signal-to-noise ratio (SNR) is $\eta =P/n_{0}=P$.

### 3.3. JSCC-Cast: Analog Decoder and Receiver

**Figure 2:**
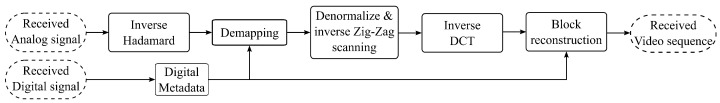
Block diagram of the JSCC-Cast receiver and decoder.

The operations at reception to recover the transmitted video sequence are shown in Figure 2. They basically consist of the inverse operations carried out at the transmitter side. Indeed, the first step is the application of the inverse Hadamard transform, and then the received symbols are decoded.

When considering linear mappings, all transformations at transmission are linear. Hence, the reception operations can be implemented jointly with a linear minimum mean square error (MMSE) estimation of the received symbols under the assumption of a Gaussian source distribution. In this case, the transmitted symbols estimating $\hat{X}$ are computed as follows:

(11) $$\begin{array}{c} \hat{X}={\bar{H}}_{n}^{T}{\left({\bar{H}}_{n}{\bar{H}}_{n}^{T}+n_{0}I\right)}^{-1}\hat{Y}, \end{array}$$

where the superindex ${}^{T}$ denotes matrix transposition, and the following is the case:

(12) $${\bar{H}}_{n}=\sqrt{P}\cdot H_{n},$$

where $H_{n}$ is the Hadamard matrix of order *n*. Note that the estimates of the vectors ${\bar{s}}_{j}$ can be directly obtained from $\hat{X}$ by inverting the scaling factor $\sqrt{\lambda }$ in (6).

When the vectors ${\bar{s}}_{j}$ are encoded with a nonlinear mapping, a more sophisticated demapping operation is necessary to determine the transmitted symbols. From the matrix $\hat{X}$, we first have to separate the transmitted symbol estimates corresponding to each vector $x_{j}$, which we will denote as

(13) $${\hat{x}}_{j}=\left[{\hat{x}}_{1,j},\ldots ,\ldots ,{\hat{x}}_{N_{j},j}\right].$$

Since analog encoding is being considered, the optimal demapping is the MMSE estimation of ${\bar{s}}_{j}$ from the received symbols ${\hat{x}}_{j}$. However, the complexity of this operation is quite high due to the nonlinear nature of the mapping function in Equation (7). For this reason, it is preferable to consider a suboptimal strategy with a much lower complexity. Following the same idea proposed in [30] for the Archimedean spiral, we consider maximum likelihood (ML) demapping where the transmitted symbols are estimated as follows.

(14) $$\begin{array}{c} {\tilde{s}}_{i,j}=\arg\max_{r}\;\;p\left({\hat{x}}_{i,j}|r\right)=\arg\min_{r}{\left\|{\hat{x}}_{i,j}-f_{j}\left(r\right)\right\|}^{2}. \end{array}$$

As observed, the above equation simply chooses the symbol *r* that minimizes the Euclidean distance between the vector of encoded symbols $f_{j}\left(r\right)$ and the corresponding vector of received symbols ${\hat{x}}_{i,j}$. Finally, the entire vector of estimated symbols for ${\bar{s}}_{j}$ is obtained by stacking all the estimates ${\tilde{s}}_{i,j}$ into a single vector, i.e., we have the following.

(15) $${\tilde{s}}_{j}=\left[{\tilde{s}}_{1,j},\ldots ,{\tilde{s}}_{N_{j},j}\right].$$

After obtaining the estimates of the *j*-th data stream normalized symbols, the next step is to undo the power normalization and mean removal carried out in Equation (2) in order to obtain the estimates of the transformed coefficients. This is achieved as follows.

(16) $${\hat{s}}_{j}=\sigma_{j}\;{\tilde{s}}_{j}+\mu_{j}.$$

The last step at the decoder is to reconstruct each B×B×F 3D array from the vectors ${\hat{s}}_{j}$, filling with zeros those elements corresponding to the coefficients removed at the transmitter. Finally, the inverse of the considered transform is individually applied to each 3D array in order to reconstruct the *F* frames of the video sequence.

### 3.4. Evaluation Metrics

After decoding the input video at the receiver, we are interested in evaluating the performance of JSCC-Cast in terms of the amount of required metadata and the quality of the reconstructed video.

The structural similarity index measure (SSIM) [31] and the peak signal-to-noise ratio (PSNR) [32] are considered to assess the quality of the reconstructed video. The first one is the most similar to the subjective mean opinion scores (MOS) [33], whereas the second one is the most frequently considered metric in the literature [34] and is based on the computation of the mean square error (MSE) [35] between the pixels of the original and the decoded frame sequences, i.e., we have the following:

(17) $$\begin{array}{c} \mathrm{MSE}=\frac{1}{NMF}\sum_{i=1}^{N}\sum_{j=1}^{M}\sum_{k=1}^{F}{\left(A_{i,j,k}-B_{i,j,k}\right)}^{2}, \end{array}$$

where A is the original video sequence, B contains the decoded frames, *N* and *M* represent the spatial dimensions of the considered video sequence, and *F* is the number of frames. The PSNR is defined from Equation (17) as follows:

(18) $$\begin{array}{c} \mathrm{PSNR}=10\log_{10}\left(\frac{{\mathrm{MAX}}_{I}^{2}}{\mathrm{MSE}}\right), \end{array}$$

where ${\mathrm{MAX}}_{I}$ represents the maximum value that a pixel may have and is given by the bit depth. We consider this metric for comparison with previous systems available in the literature.

The SSIM is an image metric that evaluates the distortion of luminance, contrast, and image structure. The SSIM is defined as follows:

(19) $$\mathrm{SSIM}\left(x,y\right)=\frac{\left(2\mu_{x}\mu_{y}+C_{1}\right)\left(2\sigma_{xy}+C_{2}\right)}{\left(\mu_{x}^{2}+\mu_{y}^{2}+C_{1}\right)\left(\sigma_{x}^{2}+\sigma_{y}^{2}+C_{2}\right)},$$

where *x* and *y* are two image signals, μ is the signal mean, and σ is the standard deviation of the signal:

(20) $$\sigma_{xy}=\frac{1}{N-1}\sum_{i=1}^{N}\left(x_{i}-\mu_{x}\right)\left(y_{i}-\mu_{y}\right),$$

(21) $$C_{1}={\left(K_{1}L\right)}^{2},$$

(22) $$C_{2}={\left(K_{2}L\right)}^{2},$$

with *L* being the dynamic range of the image pixel value, and $K_{1}\ll 1$ and $K_{2}\ll 1$ are constants.

The SSIM is fairer than the PSNR because it aims at approximating the perceptual quality of images by modeling some of the characteristics of the human visual system. Since the SSIM is a metric designed for images, it must be applied frame by frame. In order to handle a representative single value, an arithmetic mean is applied to the set of individual SSIM values obtained for the different frames.

## 4. System Design

This section details and justifies the design decisions for JSCC-Cast analog video encoding and transmission for which its main signal processing tasks are shown in Figure 1 and Figure 2.

### 4.1. Domain Transforms for Images and Video

Domain transforms are intended to concentrate most of the energy present in a video frame and relevant to the human perception into a few coefficients. Therefore, these transforms facilitate the elimination of the coefficients less significant to the human eye and allow for low-bandwidth ratios while preserving perceived quality.

There is a wide variety of transforms for the compression of video frames and sequences which are employed in both digital and analog systems. The most well known, due to their properties and characteristics, are the discrete Fourier transform (DFT), the DCT, the Walsh–Hadamard transform (WHT), and the DWT. Each of them presents a different response to compression and also on the robustness against noise [35]. The DCT appears as the transform that offers the best behavior in noisy environments when the output is truncated, thus eliminating the less significant values for the human eye.

**Figure 3:**
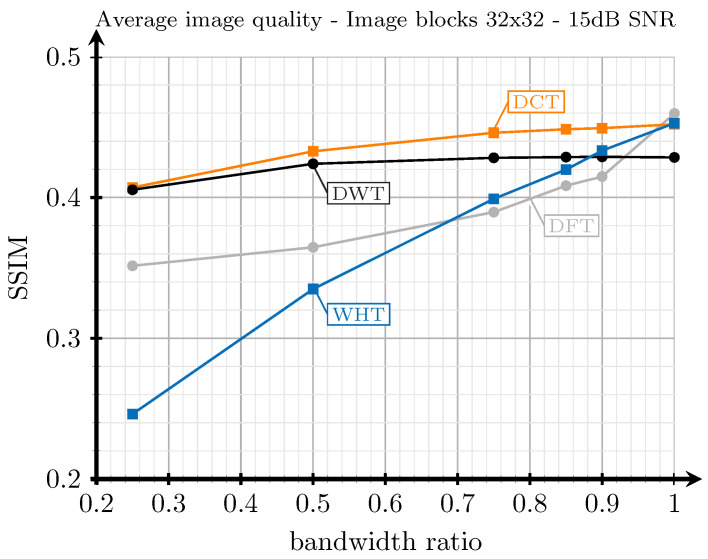
Relationship between bandwidth ratio (see Equation (5)) and image or video frame quality (SSIM) in an AWGN channel with an SNR of 15 dB [36]. In the chart, the orange line corresponds to the DCT, the black one to the DWT, the grey one to the DFT, and the blue one to the WHT.

In our previous work [36], we analyzed the performance of image and video frames transforms with compression and over noisy channels. We have performed a similar analysis for 13 still images extracted from [37] with respect to the bandwidth ratio for an AWGN channel with an SNR of 15 dB and considering that the images are split into blocks of size 32 × 32 (i.e., B=32). The average results for the obtained SSIM are shown in Figure 3. In this case, the DCT exhibits the best performance for most of the bandwidth ratio values. Only the DFT and WHT slightly outperform the DCT when the compression is negligible. In view of these previous results for still images, the DCT has been chosen for JSCC-Cast.

### 4.2. Block Spatial Division of the Video Frames

The transforms can be applied to the whole image or to regions of it. Hence, one of the main issues is the selection of the size of the region or block for which the transform will be applied to. In [35], there is a performance analysis about the most common transforms used in image compression according to their division into blocks and truncating 75 % of the output coefficients. The results show that the optimal block sizes are 512 × 512 for the DFT, 16 × 16 or 32 × 32 for the DCT, and 16×16 for the WHT. Based on these results and in order to minimize the amount of metadata, the block size chosen is 32×32 pixels (i.e., B=32).

### 4.3. Temporal Redundancy

Source video is a temporal sequence of frames. Therefore, video compression is possible by individually applying a 2D-DCT to each video frame, in which case only the spatial correlation is exploited. It is possible to increase the compression level by considering the temporal correlation between contiguous frames. In the particular case of the DCT, this results in the 3D-DCT, which is mathematically defined as the following [38]:

(23) $$\begin{array}{cc} \; & S\left(u,v,w\right)=\;\alpha_{3D}\left(u,v,w\right)\;\cdot \sum_{x=0}^{N_{C}-1}\sum_{y=0}^{N_{R}-1}\sum_{z=0}^{N_{F}-1}s\left(x,y,z\right)\cos\left(t_{1}\right)\cos\left(t_{2}\right)\cos\left(t_{3}\right) \end{array}$$

where

(24) $$\begin{array}{c} \alpha_{3D}\left(u,v,w\right)=\sqrt{\frac{2}{N_{R}}\frac{2}{N_{C}}\frac{2}{N_{F}}}C\left(u\right)C\left(v\right)C\left(w\right) \end{array}$$

with

$$C\left(k\right)=\left\{\begin{array}{cc} \frac{1}{\sqrt{2}} & k=0\\ 1 & \mathrm{otherwise} \end{array}\right.$$

and

$$\begin{array}{c} t_{1}=\frac{\left(2x+1\right)u\pi }{2N_{C}},\;t_{2}=\frac{\left(2y+1\right)v\pi }{2N_{R}},\;t_{3}=\frac{\left(2z+1\right)w\pi }{2N_{F}}, \end{array}$$

where $N_{C}$, $N_{R}$, and $N_{F}$ represent the size of the 3D block, s denotes the input symbols, and S denotes the transformed output symbols. In a similar manner, the inverse transform for recovering the original symbols is given by the following.

(25) $$\begin{array}{cc} \; & s\left(x,y,z\right)=\;\alpha_{3D}\left(u,v,w\right)\;\cdot \sum_{u=0}^{N_{C}-1}\sum_{v=0}^{N_{R}-1}\sum_{w=0}^{N_{F}-1}\;S\left(u,v,w\right)\cos\left(t_{1}\right)\cos\left(t_{2}\right)\cos\left(t_{3}\right). \end{array}$$

As is observed, the 3D-DCT takes into account the temporal dimension, $N_{F}$, in order to build the input blocks. We have decided to work with sets of 8 frames, thus considering blocks of size 32×32×8. Assuming that the video sequences are recorded at 25 frames per second, this number is reasonable since the system needs several frames to obtain the benefit of exploiting the temporal redundancy. However, the number of frames cannot be too large to avoid introducing a significant transmission delay.

In general, 3D-DCT shows a superior performance with respect to 2D-DCT in terms of video compression. However, we will also consider a mixed strategy which consists in selecting the best approach for each block. In this case, the decision of applying the 2D or the 3D DCT to one specific block is made on the basis of which approach provides the lowest MSE. The mixed strategy works as follows. First, both types of DCT are applied followed by the decimation procedure. Next, the inverse DCT is applied, and the MSE between the original and the restored block is determined for both the 2D and the 3D-DCT. Finally, the option that exhibits the lowest MSE value is the one that is chosen.

**Figure 4:**
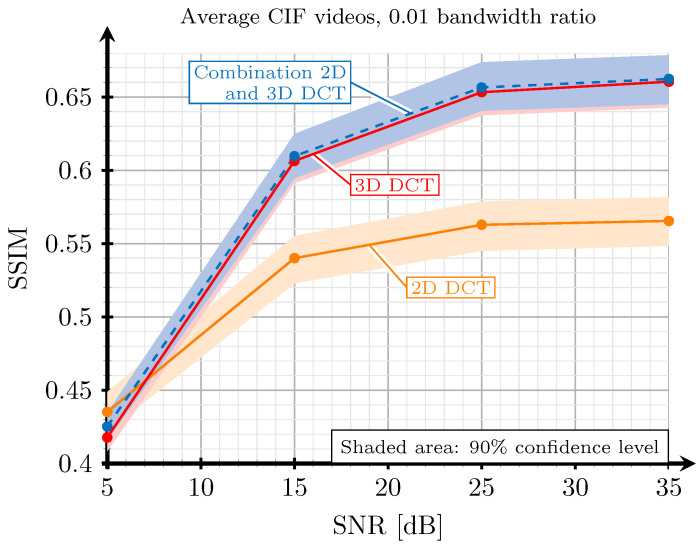
Relationship between channel SNR and the SSIM obtained with 2D-DCT (orange line), 3D-DCT (red line), and the selection of the best DCT for each block (blue line).

**Figure 5:**
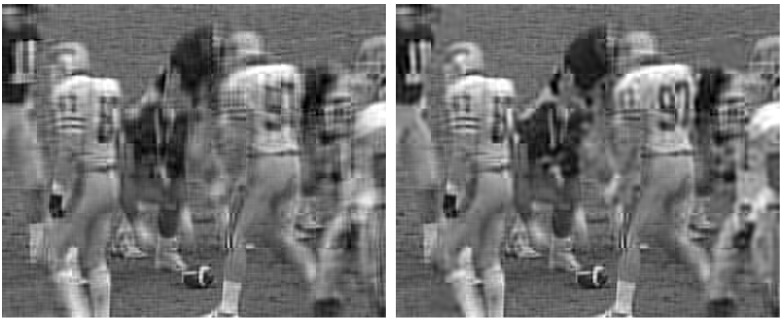
Visual comparison of 3D-DCT (**left**) vs. a combination of 2D and 3D-DCT (**right**) with the JSCC-Cast analog video system. The left image has SSIM = 0.61 and PSNR = 21.81 dB. The right one has SSIM = 0.63 and PSNR = 22.25 dB. The bandwidth ratio is set to 0.01, and the channel SNR is 30 dB. Frame extracted from the transmission of the video “football”.

Figure 4 shows the obtained SSIM when applying the 2D-DCT, the 3D-DCT, and the best one between both for different SNR values in an AWGN channel and a bandwidth ratio of 0.01. The results were obtained by averaging all the SSIM values from each frame of the “football” video [39]. As a conclusion from these results, we can state that the combination of 2D and 3D-DCT is the choice that achieves the best image quality. Although the gain in terms of SSIM is not very high compared to the 3D-DCT, it should be noted that, in certain areas of the frames where there is a lot of movement, the visual quality will be higher when using the 2D-DCT. Hence, the result of combining the 3D-DCT for static areas and 2D-DCT for motion ones results in video sequences with a better perceptual quality in general. In Figure 5, it is possible to observe this effect with an exemplary frame. The numbers of the back of player 97 are clearly displayed at the output of the combination of 2D and 3D-DCT, whereas the numbers appear blurred when using only the 3D-DCT. This part of the frame corresponds to an area in motion in the considered video.

It is important to note that the use of two transforms increases the required metadata in one bit per block. If we want to reduce the metadata as well as the system complexity to the minimum, the best choice is to consider only the 3D-DCT. In this work, however, we have decided that the small additional amount of metadata required by the mixed approach is acceptable considering the benefits in terms of perceived quality.

### 4.4. Frequency Coefficients Rearrangement Pattern

For both the 2D and 3D-DCT, the source video is split into frames of 32 × 32 pixels. In the 2D case, DCT is applied frame by frame individually, whereas in the 3D case, eight consecutive frames are considered, i.e., the 3D DCT is applied to blocks of 32 × 32 × 8 pixels. After the DCT transform, the coefficients of every single block are rearranged into a vector following a zig-zag pattern for the 2D case and an hyperboloid [40] for the 3D case. This step is intended to sort the transformed coefficients according to their frequency, i.e., from lower to higher frequencies. Next, compression is achieved by removing a portion of the symbols corresponding to the highest frequencies. Note that the number of coefficients which are selected/removed depends on the desired bandwidth ratio (see Equation (5)).

At reception, the positions of the received vector corresponding to the removed symbols are filled with zeros. Next, the vector is reordered into a matrix using the inverse zig-zag or the inverse hyperboloid. Finally, the appropriate inverse of the DCT is applied.

The choice of the rearrangement pattern is an important issue as it impacts the quality of the encoded video. Therefore, it is necessary to evaluate how the symbols are rearranged at the output of the DCTs in order to correctly select the order of the symbols in the frequency domain. The zig-zag pattern is the optimal choice for the 2D-DCT. However, for the 3D-DCT, there is a wide range of options available in the literature such as the isoplane [41,42], the hyperboloid [40,43], and even dividing in zones the 3D array with the frequency coefficients [44]. All of these strategies for rearranging the symbols in the transform domain are based on the fact that the elements closer to the DC element are the ones that carry the most valuable information for the human eye, whereas those that are more distant can be discarded as they have minor impact on the perceived quality. We focus on the first two options, isoplane and hyperboloid, and discard the division into zones because it is not flexible enough in terms of bandwidth ratios and 3D array sizes since complete areas need to be transmitted.

#### 4.4.1. Isoplane

**Figure 6:**
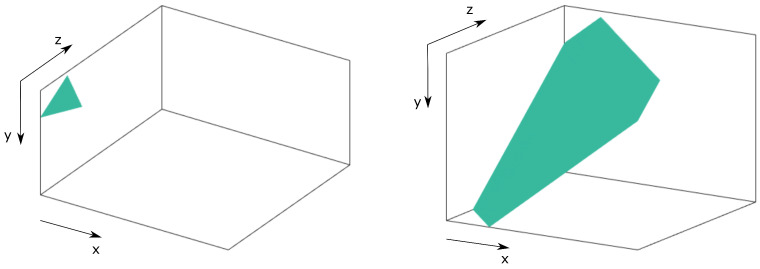
Example of isoplane layers for a 32×32×32 symbol 3D array. Left hand-side graph for K=10 and right hand-side graph for K=40.

This method sorts the 3D-DCT symbols according to the sum of their position indices [41,42], i.e., we have the following:

(26) g(x,y,z)=x+y+z=K,

where *x*, *y*, and *z* represent the coordinates of the coefficients satisfying z≤y≤x, and *K* is a constant representing the plane. Figure 6 shows an example of the way in which the layers of the cube are traversed following Equation (26), providing a similar route to the zig-zag pattern but incorporating the temporal dimension.

#### 4.4.2. Hyperboloid

**Figure 7:**
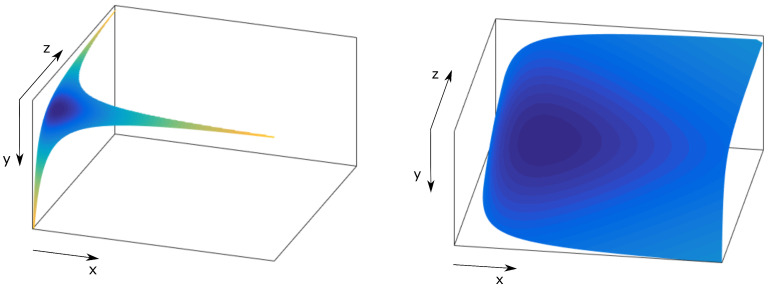
Example of hyperboloid layers for a 32×32×32 symbol 3D array. Left hand-side graph for K=40 and right hand-side graph for K=100.

This method of rearranging the symbols at the output of the 3D-DCT is an improvement over the isoplane-based approach [40,43]. The aim is to optimize the selection of symbols according to their proximity to the DC value. This strategy can be formulated as follows:

(27) $$\begin{array}{c} g(x,y,z)=xyz=K, \end{array}$$

with *K* a constant and z≤y≤x. Hence, this method consists in selecting layers according to the importance of the value which results from the product of their Cartesian coordinates. It starts at (0,0,0) for the DC symbol, which is the most important one, and finishes at the opposite end of the 3D array located at (32,32,8). Note that the layers will have different shapes depending on their level. Two examples of layers selected as hyperboloids are shown in Figure 7.

#### 4.4.3. Analysis of the Rearranging Methods

**Figure 8:**
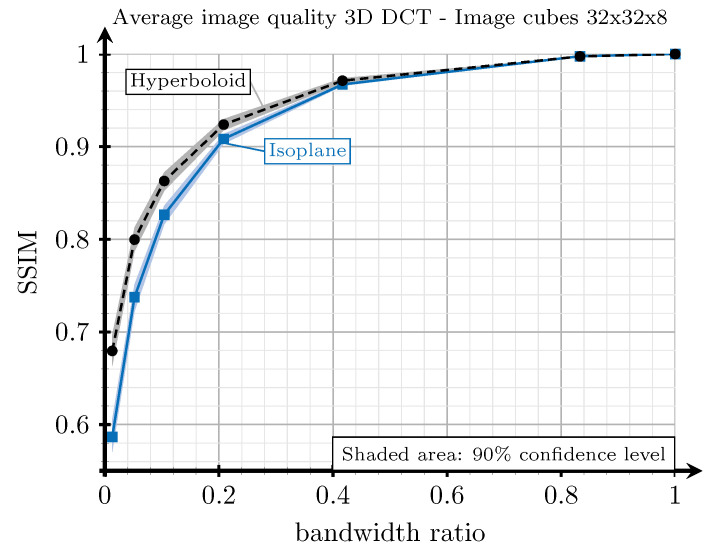
SSIM versus bandwidth ratio for the isoplane (blue line) and the hyperboloid (black dashed line) strategies to arrange the coefficients at the output of the 3D-DCT. The results are averaged considering four videos extracted from [39].

Figure 8 shows the results of the experiments carried out to analyze the performance of the isoplane and the hyperboloid strategies for different bandwidth ratios. The SSIM index was computed by averaging the values obtained for several videos extracted from [39]. As observed, these results confirm the better behaviour of the hyperboloid approach for all bandwidth ratios, although the performance gains vanish as the bandwidth ratio increases (lower compression scenarios).

Figure 8 also shows the relationship between compression and image quality achieved with the 3D-DCT. We can say that SSIM values above 0.9 correspond to excellent video qualities and that increasing the bandwidth ratio above 0.2 does not provide significant improvements in quality.

### 4.5. Redundancy Analysis

As mentioned in Section 3, the DCT output is split into two separate data streams corresponding to the DC and AC coefficients, respectively. Since the impact of these two types of coefficients on the visual quality of the reconstructed video is very different, JSCC-Cast considers a different protection level for each of them. In particular, redundancy is introduced only for the DC coefficients, whereas the AC coefficients are just linearly encoded. This decision is supported by the fact that DC coefficients usually convey the most significant visual perceived information. Hence, protecting the DC coefficients with redundancy will result in the quality improvement of the reconstructed frames when transmitting over noisy channels [4].

**Figure 9:**
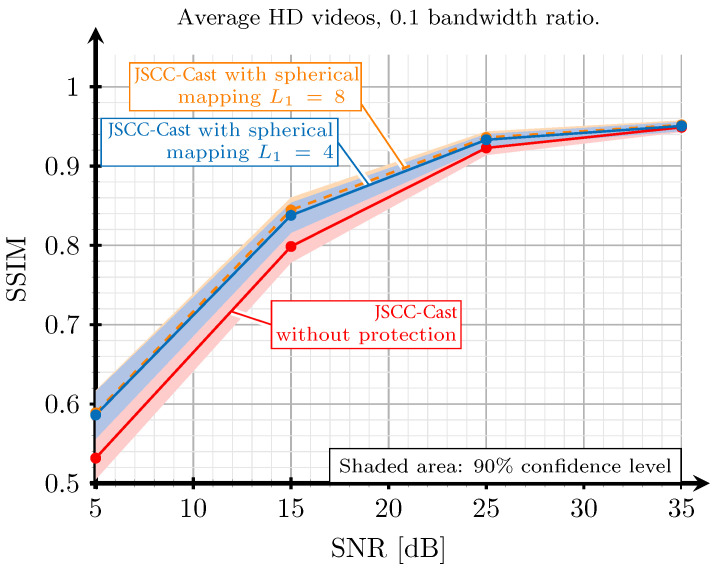
SSIM versus SNR obtained by JSCC-Cast considering three different redundancy strategies to protect the DC coefficients. The best combinations of 2D and 3D-DCT are used for a bandwidth ratio of 0.1. The red line corresponds to transmission without protection, the blue line to a spherical mapping with redundancy $L_{1}\;=\;4$, and the dashed orange line to a spherical mapping with redundancy $L_{1}\;=\;8$.

Figure 9 shows the performance of the proposed JSCC-Cast encoding scheme when DC coefficients are encoded by using the orthogonal spherical mappings defined in (7) and considering two expansion factors, $L_{1}\;=\;4$ and $L_{1}\;=\;8$. The obtained results are compared to the performance of a linear encoding of such coefficients ($L_{1}\;=\;1$). This experiment has been carried out by considering the system configuration that mixes 2D and 3D-DCT for a bandwidth ratio of 0.1. Figure 9 shows an improvement when using spherical mappings for the whole SNR range, although this performance gain is more remarkable for low and medium SNR values. Moreover, the spherical encoding with the two expansion factors ($L_{1}\;=\;4$ and $L_{1}\;=\;8$) exhibits a similar performance. Hence, $L_{1}\;=\;4$ was chosen for JSCC-Cast as it has the advantage of transmitting less redundant symbols. In this case, the amount of redundancy introduced with the use of spherical codes does not considerably increase the bandwidth ratio for the analog encoded video. Recall that video encoding operations are applied to blocks of 32×32×8 pixels, i.e, there are 8192 coefficients per block after the transformation step. When applying the 3D DCT, we obtain a single DC coefficient; therefore, only three additional redundant symbols are introduced. However, when applying the 2D-DCT, the number of DC coefficients increases up to eight (one per frame). Hence, the additional symbols become 24, which is still very low compared to the total number of symbols.

## 5. Evaluation and Results

In this section we present the results obtained from different computer experiments carried out in order to evaluate the performance of JSCC-Cast. As a benchmark, we consider Softcast [8], an analog video system that actually uses digitally encoded metadata and that is the most used one as a benchmark in the literature [9,10,11,12,13,14,15,16,18,19,20,21,22,23,24,25,45,46]. In the performance comparison, we also included H.265 as a state-of-the-art, all-digital video system. An SNR range between 5 dB and 35 dB has been selected to test and compare the system in a range similar to that of real situations. Such an SNR range is similar to those of the works cited above. It is interesting to note that in low power consumption systems, such as IoT, SNR levels can be below 20 dB.

### 5.1. Digital Implementation

As stated above, the proposed JSCC-Cast system will be compared with an all-digital system based on the H.265 standard, one of the most advanced digital video codecs. However, the comparison methodology is not trivial because of the different characteristics of both schemes, but we can say that the proposed system is simpler to implement than H.265 for several reasons such as the number of signal processing tasks required by H.265 [47]. Additionally, most digital transmission systems require a feedback channel from the receiver to the sender, which is unnecessary in our proposed system.

The design philosophy of the H.265 is significantly different than that of JSCC-Cast because JSCC-Cast aims at providing an acceptable video quality for a wide range of situations requiring a negligible amount of critical metadata, whereas the all-digital system focuses on providing as much quality as possible for a particular bandwidth ratio, which needs to be adapted depending on the channel conditions. In this sense, the all-digital implementation should be interpreted as an upper bound where most of the limitations considered for the design of the analog scheme are relaxed.

As an implementation of the H.265 codec, we have used the x265 library [48] by means of the the FFmpeg software [49]. In this case, the video output is configured to have a constant bit rate (CBR). The actual value of the bitrate will depend on the symbol rate at the output of JSCC-Cast and the channel conditions. From the rate of the symbols transmitted by the JSCC-Cast and considering the configuration of the digital system necessary in order to guarantee an error-free transmission, we determine the number of bits per second that the digital system should employ in order to encode the video sequence. The number of modulation levels is adjusted according to Table 1, for which its values were extracted from the results available in [50]. For the sake of simplicity, the channel coding rate is set to 1/2.

Another issue is the practical implementation of the digital H.265-based system. JSCC-Cast was designed to operate on small blocks of frames, but the all-digital system requires exploiting greater temporal redundancy in order to perform correctly. Therefore, in the all-digital system, video sequences are processed in blocks of 25 frames.

### 5.2. Evaluation Based on SSIM and PSNR

We consider two different types of video sequences depending on their resolution: common intermediate format (CIF) and full high-definition (HD). We have also considered two particular bandwidth ratios: 0.05 and 0.1 for CIF videos, and 0.005 and 0.01 for full HD videos. This decision is because of the fact that similar average SSIM values were obtained for both resolutions with these rates; hence, it is sensible to compare their behaviour. The performance obtained with JSCC-Cast is compared to that provided by Softcast and with the all-digital system described in Section 5.1.

**Figure 10:**
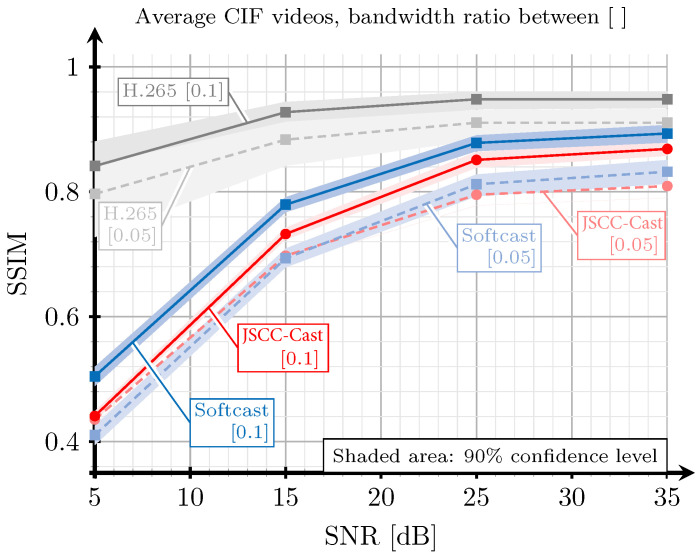
SSIM versus SNR for a selection of videos with CIF resolution extracted from [39]. The red lines correspond to the proposed JSCC-Cast, the blue lines to Softcast, and the grey lines to H.265. The solid lines correspond to a bandwidth ratio of 0.1, and the dashed lines correspond to a bandwidth ratio of 0.05.

**Figure 11:**
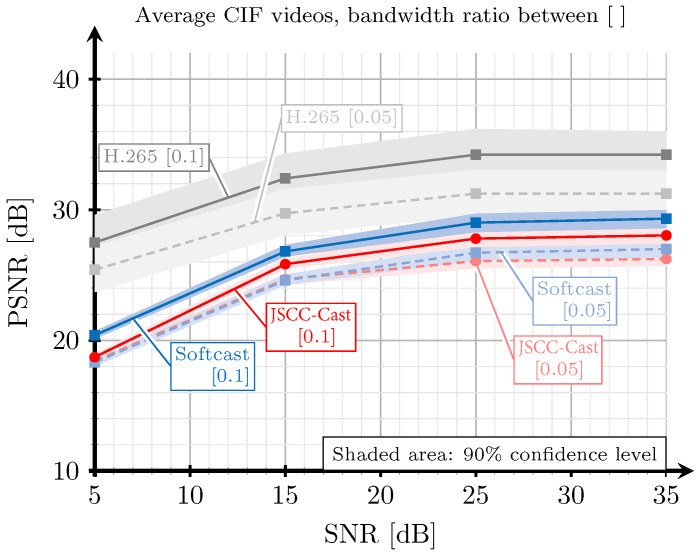
PSNR versus SNR for a selection of videos with CIF resolution extracted from [39]. The red lines correspond to the proposed JSCC-Cast, the blue lines to Softcast, and the grey lines to H.265. The solid lines correspond to a bandwidth ratio of 0.1, and the dashed lines correspond to a bandwidth ratio of 0.05.

Figure 10 plots the image quality in terms of SSIM with respect to the channel SNR for the three systems to be compared: the proposed JSCC-Cast, Softcast, and the H.265-based all-digital system. Recall that the SNR is measured as $\eta =P/n_{0}$, i.e., the quotient between the transmit power and the noise variance. These results were obtained considering a selection of videos with CIF resolution extracted from [39] transmitted over an AWGN channel with two bandwidth ratios: 0.05 and 0.1. This simulation parameter determines the number of transformed coefficients selected for each 3D array in the compression phase according to Equation (5). As detailed in Section 5.1, the bit rate of the H.265-based system also depends on the bandwidth ratio and the constellation selected according to the channel noise level. The results for the same scenario, although considering the PSNR metric, are shown in Figure 11. As observed, these results are very similar to those obtained for the SSIM index in Figure 10.

According to the results shown in Figure 10 and Figure 11, the JSCC-Cast achieves a near performance to that of Softcast for the two considered bandwidth ratios. For the highest ratio, Softcast provides higher SSIM values for all channel SNR ranges, although these gains are small. For the lowest ratio, the JSCC-Cast is even able to outperform Softcast in the low channel SNR regime. This is related to the protection of the DC coefficients introduced by the proposed system, which mitigates the impact of the channel noise on these coefficients. In addition, low bandwidth ratios imply that more high-frequency information from the frames is removed prior to the encoding operation; therefore, the importance of the low-frequency coefficients is more perceptible. This result is especially interesting because Softcast is typically employed as the benchmark for analog video encoding. In addition, recall that the amount of metadata required by the JSCC-Cast is significantly smaller, as we will show in Section 5.4.

Regardless of the comparison with the all-digital system, the H.265 encoder always outperforms its analog counterparts, especially for low SNR values. Such a performance difference becomes smaller as the channel SNR increases. This behavior was expected since the quality of the received videos with analog transmissions directly depends on the channel quality, whereas the digital transmission shows a floor effect. As mentioned, this behavior of the analog video schemes is particularly attractive for multicast scenarios.

Although the all-digital scheme provides the best performance, it is important to emphasize that the comparison between the analog-based approaches and the all-digital one is not utterly fair. On the one hand, JSCC-Cast is simpler than the all-digital one since it requires less signal processing tasks and no feedback. For this reason, this encoding scheme is specially suitable for devices with restricted computational resources such as, for example, IoT devices where power consumption is a fundamental issue. On the other hand, real-world wireless channels, contrarily to simulated AWGN channels, exhibit fluctuations (the so-called fading) that penalize all-digital systems:

- A feedback link between the receiver and the transmitter is required to inform the transmitter about the channel quality. According to this information, the transmitter adapts its modulation and coding scheme, resulting in slower transmissions when the channel quality drops, hence reducing the overall system performance.
- In the case of deep channel fading situations, it is very likely that the digital system transmits above the channel capacity, especially if the channel fluctuates fast over time. This results in a situation where the receiver cannot recover the transmitted data, hence requiring additional strategies such as retransmissions, which severely penalize the throughput.

In contrast to all-digital systems, JSCC-Cast does not need to track channel variations. This greatly simplifies the design of the encoding-transmission operations and reduces the overall complexity. In view of the results and considering the general characteristics of the analog-domain processing, we can conclude that there will be certain scenarios in which the performance of the proposed JSCC-Cast is reasonably good and is a better choice than all-digital systems.

**Figure 12:**
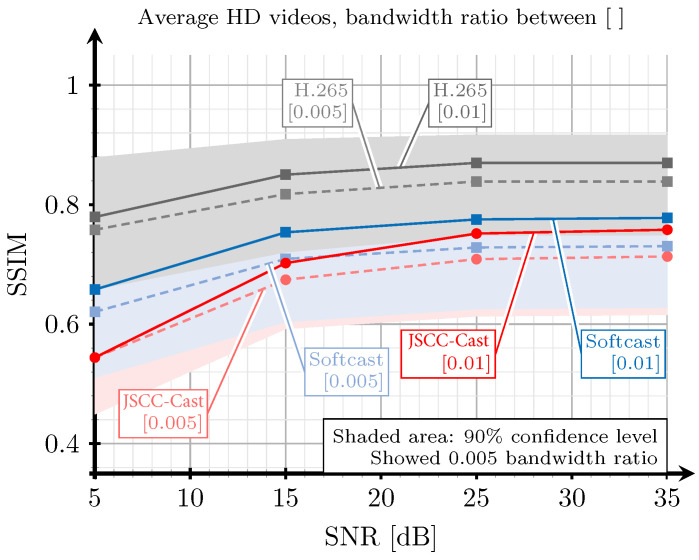
SSIM versus SNR for a selection of videos with full HD resolution extracted from [39]. The red lines correspond to the proposed JSCC-Cast, the blue lines to Softcast, and the grey lines to H.265. The solid lines correspond to a bandwidth ratio of 0.1, and the dashed lines correspond to a bandwidth ratio of 0.05.

Figure 12 shows the SSIM obtained for the same three video systems considering now a selection of full HD videos extracted from [39] and the corresponding bandwidth ratios for the full HD resolution. As observed, the behavior of the SSIM curves is similar to those observed for the case of a CIF resolution. Although Softcast provides average better results, the video quality with the proposed JSCC-Cast is quite similar and the confidence intervals actually overlap. Therefore, no statistically significant performance differences between the two systems can be concluded.

**Figure 13:**
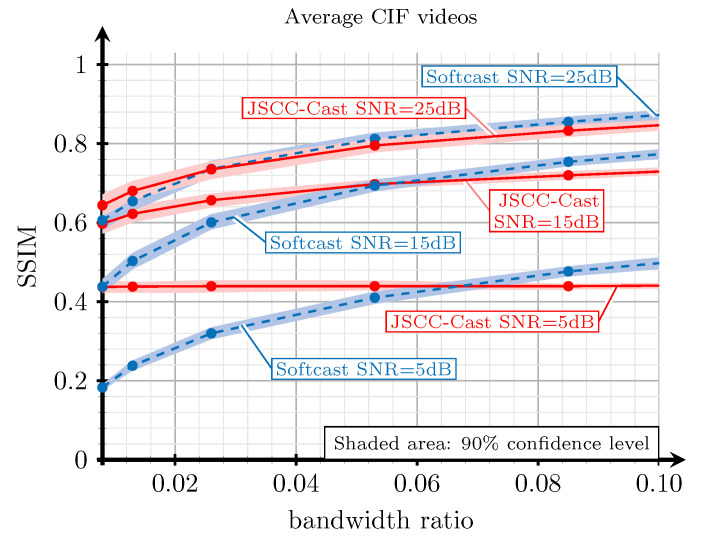
SSIM versus bandwidth ratio for a selection of videos with CIF resolution extracted from [39]. The SSIM values were obtained with the proposed JSCC-Cast video system and Softcast for different channel qualities. The red lines correspond to the proposed JSCC-Cast, and the blue lines correspond to Softcast.

Another interesting comparison between the proposed JSCC-Cast and Softcast consists in analyzing their performance with respect to the compression level applied to the source videos for a given channel noise level. Figure 13 presents the SSIM obtained with both schemes for the desired range of bandwidth ratios considering CIF videos and several channel SNR values. As observed, the proposed analog scheme provides significant gains for low bandwidth ratios and for medium and low channel SNR values, this behavior is especially interesting for systems with severe constraints on the power consumption, such as IoT applications where the practical SNR values could be below 20 dB. In general, JSCC-Cast is able to closely approach the performance of Softcast for high video qualities (SSIM values), whereas it clearly outperforms Softcast when the observed quality of the received video decreases. Thus, we conclude that the proposed analog video system presents good performance for a practical range of bandwidth ratios.

Another remarkable result from the previous experiment is the flat behavior exhibited by the JSCC-Cast regardless of the bandwidth ratio for a channel SNR of 5 dB. This indicates that the quality of the transmitted videos in the low SNR regime is essentially dominated by the particular encoding of the DC coefficients. Thus, increasing the number of AC coefficients has a minimum impact on the quality of the reconstructed videos because they are received with too much noise. As a consequence, they do not contribute in refining the frame’s details.

### 5.3. Perceptual Evaluation Based on Visual Comparison

In this section, we illustrate the performance of the two considered analog schemes (the proposed one and Softcast) by means of a visual comparison with some video frames extracted from the computer experiments. This visual analysis aims at illustrating that the perceptual quality of the videos transmitted with both systems is eventually similar in spite of the SSIM or PSNR differences presented in the previous figures.

**Figure 14:**
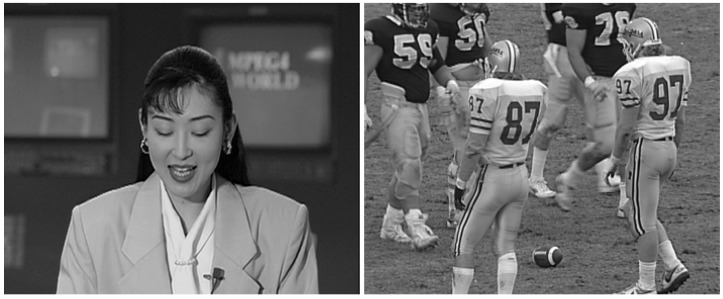
**Left-hand side**: “Akiyo” original frame. **Right-hand side**: “Football” original frame. Videos extracted from [39].

**Figure 15:**
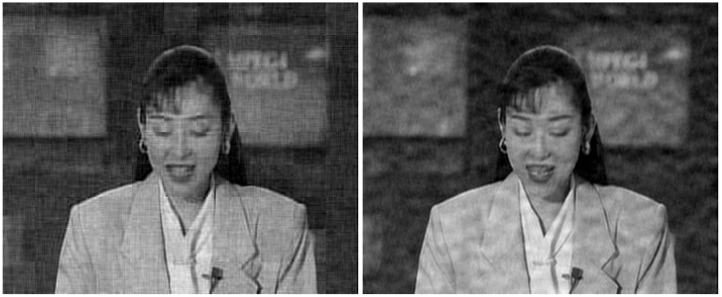
“Akiyo” frames received from a transmission with a bandwidth ratio of 0.1 and a SNR of 10 dB. **Left-hand side**: JSCC-Cast with SSIM quality of 0.67. **Right-hand side**: Softcast with SSIM quality of 0.76.

**Figure 16:**
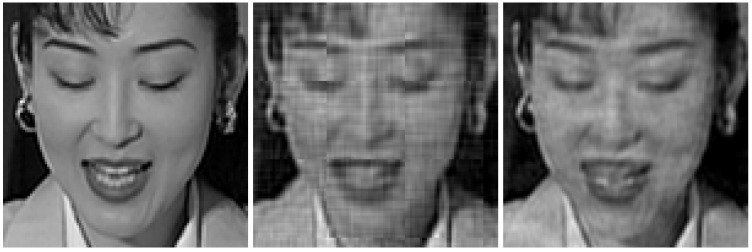
Zoom detail from Figure 15. “Akiyo” frames received from a transmission with a bandwidth ratio of 0.1 and a SNR of 10 dB. **Left-hand side**: original frame. **Center**: JSCC-Cast with SSIM quality of 0.67. **Right-hand side**: Softcast with SSIM quality of 0.76.

**Figure 17:**
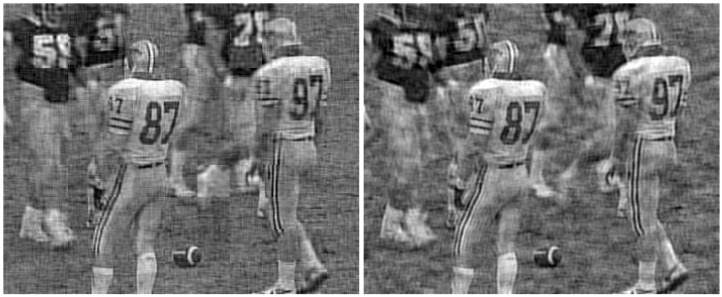
“Football” frames received from a transmission of a video with a bandwidth ratio of 0.1 and SNR=10 dB. **Left-hand side**: JSCC-Cast with SSIM quality of 0.66. **Right-hand side**: Softcast with SSIM quality of 0.66.

**Figure 18:**
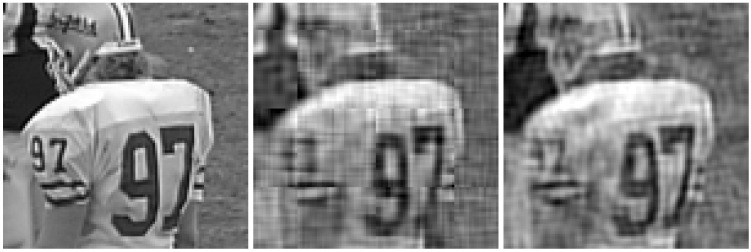
Zoom detail from Figure 17. “Football” frames received from a transmission of a video with a bandwidth ratio of 0.1 and SNR=10 dB. **Left-hand side**: original. **Center**: JSCC-Cast with SSIM quality of 0.66. **Right-hand side**: Softcast with SSIM quality of 0.66.

The considered original frames are shown in Figure 14, whereas the same frames encoded with the proposed JSCC-Cast and transmitted over a channel with an SNR=10 dB and a bandwidth ratio of 0.1, are shown in Figure 15 (left-hand side) with zoom detail in Figure 16 and Figure 17 (left-hand side) with zoom detail in Figure 18. If we compare both images, we appreciate a slight quality loss but without missing any relevant detail in the received frame. Figure 15 (right-hand side) and Figure 17 (right-hand side) show the same frames received using Softcast (zoom detail in Figure 16 and Figure 18). As observed, although the SSIM values for the frames transmitted with Softcast are equal or higher, the perception qualities are equivalent. It is of interest to show how channel noise is affected by noise intensities typical of real channels, as it is shown how it affects the picture quality in these analog systems. For the case of Softcast, homogeneous wave patterns are visible throughout the whole frame, which is due to the application of the DCT to the entire frame. Contrarily, the proposed JSCC-Cast compartmentalizes errors because of the block application of the DCT.

**Figure 19:**
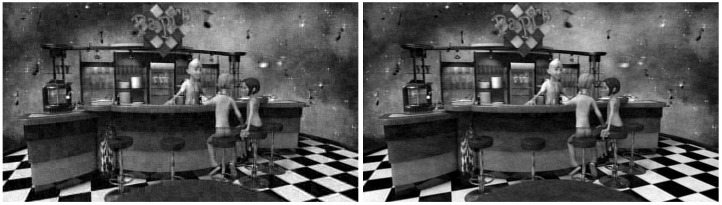
“Life” frames received from a transmission with a bandwidth ratio of 0.01 and a SNR of 15 dB. Video with full HD resolution extracted from [39]. **Left-hand side**: JSCC-Cast with a SSIM quality of 0.62. **Right-hand side**: Softcast with a SSIM quality of 0.67.

**Figure 20:**
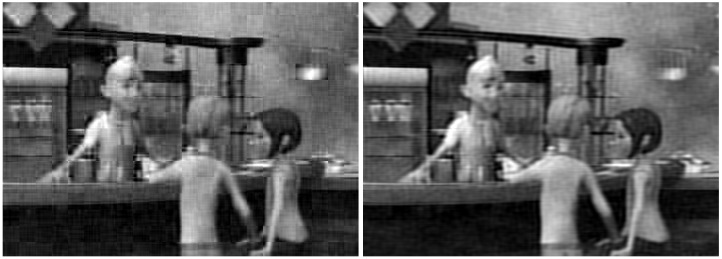
A detail from Figure 19. **Left-hand side**: JSCC-Cast with SSIM quality of 0.62. **Right-hand side**: Softcast with a SSIM quality of 0.67.

Regarding full HD videos, Figure 19 and Figure 20 show how the block edges are also perceptible with JSCC-Cast, but they are small and do not prevent the perception of details such as edges or object contours. Again, for this type of video, both video encoding and transmission systems present similar visual quality, which also confirms also the suitability of the proposed analog scheme for videos with higher resolutions.

### 5.4. Metadata Evaluation

An important issue to assess the feasibility of video coding systems is the amount of metadata required to decode the transmitted video sequences. JSCC-Cast requires the following parameters to be transmitted as metadata: a bit indicating if the block is from a 2D or 3D-DCT, the polynomial expansion parameter of the analog JSCC, and the variance and the mean from each block. Such information is critical in the decoding operation because its corruption renders the received bitstream useless. Therefore, metadata must be transmitted with an adequate level of protection to ensure an error-free transmission regardless of the channel conditions. Considering that large amounts of metadata also produce a big overhead in the transmission of the video stream, which can result in significant performance reductions, Table 2 details the amount of metadata required by JSCC-Cast and Softcast, assuming that entropy coding is applied in both cases. The same source compression scheme and redundancy levels are used for the metadata of both JSCC-Cast and Softcast.

According to Table 2, we conclude that for CIF videos, JSCC-Cast requires much less metadata than Softcast, even considering that we are transmitting with the configuration based on the best combination of 2D and 3D-DCT. These differences significantly grow with the bandwidth ratio since Softcast actually doubles the required metadata, whereas JSCC-Cast needs a similar amount of metadata regardless of the bandwidth ratio. The results in Table 2 for full HD videos show that the difference in terms of required metadata between JSCC-Cast and Softcast is even larger than for CIF videos. Hence, these results are especially interesting as they show that the amount of metadata to be encoded by JSCC-Cast is significantly smaller than in the case of Softcase, which is a considerable practical advantage.

It is worth remarking that the metadata is not included in the computation of the bandwidth ratio. This simplification is usually assumed for the evaluation of hybrid/analog schemes such as Softcast or its variations when their performance is compared to other digital alternatives. Although the volume of metadata is relatively low with respect to the volume of transmitted analog symbols, the impact of sending this information is ignored. In the case of JSCC-Cast, this simplification is actually justified, as the amount of metadata is practically negligible.

## 6. Conclusions

In this work we have proposed JSCC-Cast, an analog video encoding and transmission system that is competitive with similar contemporary hybrid systems such as Softcast and even surpassing them under certain circumstances. The paper focuses on presenting the design and optimization of JSCC-CAst for which its minimal computational cost renders it very suitable for the encoding of videos with a high resolution and a very low requirement of digital metadata. Due to its simplicity, the use of this system is oriented towards IoT devices with low power consumption and limited computational capacity.

JSCC-Cast presents a behavior similar to Softcast when considering conventional video quality metrics, such as PSNR or SSIM, for both reasonable compression levels and practical ranges of channel SNR. Indeed, the proposed analog scheme is able to outperform Softcast for low bandwidth ratios so that it is very attractive for video transmission on networks with severe bandwidth requirements. In addition, although Softcast provides slight gains in terms of the objective quality metrics, the perceived quality of the reconstructed frames is very similar. Finally, the division of the video sequences in 3D blocks allows for the application of either the 2D or 3D-DCT relative to each block, which limits the propagation of certain visual patterns on the reconstructed frames, while requiring also a minimum increase in the amount of metadata to be transmitted.

JSCC-Cast has also been compared to an all-digital system based on the H.265 standard. Under certain circumstances, such as devices with limited computational resources, time-varying wireless channels, and/or receivers with dissimilar channel conditions in multicast scenarios, the analog coding of video information is more suitable to ensure its correct visualization and reception since it achieves an acceptable performance for all considered channel SNR values.

### Future Work

Finally, we will analyze the improvements that could be made to the system to increase its performance.

- Although JSCC-Cast exhibits blocking effects in some situations, they can be mitigated at the decoder by means of smooth filtering, hence increasing the quality of video at reception.
- The performance of JSCC-Cast is relatively far from that of H.265, the state-of-the-art digital system, for a specific SNR in channels with high noise. This problem could be mitigated by applying more complex analog coding techniques in order to protect the transmitted information at the cost of increasing the computational cost of the system, without significantly increasing the amount of transmitted information.
- In analog systems the image quality is directly proportional to the noise level of the channel. In this work, we have evaluated simulated channels with AWGN noise, but it would be very interesting to deal with real channels in order to evaluate how it affects the video quality.

## Figures and Tables

**Table 1:** Relationship between the SNR and the modulation scheme that a digital system can use to transmit without errors over an AWGN channel. The data were obtained from [50].

SNR Value	Constellation
SNR ≤ 8.5 dB	BPSK
8.5 dB < SNR ≤ 13.5 dB	QPSK
13.5 dB < SNR ≤ 22 dB	16-QAM
22 dB < SNR	64-QAM

**Table 2:** Amount of metadata required by Softcast and the proposed JSCC-Cast considering the best combination of 2D and 3D-DCT (situation demanding the highest amount of metadata).

Bandwidth Ratio	Video Resolution	Bits/Frame	
		Analog	Softcast
0.05	CIF	3.9	50.8
0.1	CIF	3.8	113.6
0.2	CIF	4.0	258.6
0.005	Full HD	49.8	148
0.01	Full HD	20.4	329.7
0.05	Full HD	71.1	2106.5
0.1	Full HD	73.2	4598.6
0.2	Full HD	76.8	9974.7

## Abbreviations

The following abbreviations are used in this manuscript:

2D	Two-dimensional
3D	Three-dimensional
AC	Alternate current
AJSCC	Analog joint source channel coding
AWGN	Additive white Gaussian noise
CBR	Constant bit rate
CIF	Common intermediate format
DC	Direct current
DCT	Discrete cosine transform
DFT	Discrete Fourier transform
DWT	Discrete wavelet transform
FFT	Fast Fourier transform
FHT	Fast Hadamard transform
GMRF	Gaussian Markov random field
HD	High-definition
HEVC	High-efficiency video coding
IoT	Internet of Things
JSCC	Joint source channel coding
ML	Maximum likelihood
MMSE	Minimum mean square error
MOS	Mean opinion scores
MSE	Mean square error
PSNR	Peak signal-to-noise ratio
SNR	Signal-to-noise ratio
SSIM	Structural similarity index measure
WHT	Walsh–Hadamard transform
